# NOTCH 1 Mutation in a Patient with Spontaneous and Recurrent Dissections of Extracranial Arteries

**DOI:** 10.3389/fneur.2017.00245

**Published:** 2017-06-09

**Authors:** Carlos Guevara, Gonzalo Farias, Kateryna Bulatova, Pablo Alarcón, Wendy Soruco, Carlos Robles, Marcelo Morales

**Affiliations:** ^1^Clínica de Neurología, Servicio de Neurología y Neurocirugía, Hospital Clínico Universidad de Chile, Santiago, Chile; ^2^Sección Genética, Departamento de Medicina, Hospital Clínico Universidad de Chile, Santiago, Chile; ^3^Sección Neurorradiologia, Servicio de Imágenes, Hospital Clínico Universidad de Chile, Santiago, Chile; ^4^Clínica de Cardiologia, Hospital San Juan de Dios, Santiago, Chile

**Keywords:** NOTCH1, stroke, dissection of extracranial arteries, recurrent dissection of extracranial arteries, Pro2122Leu

## Abstract

Dissections of extracranial arteries are estimated to account for only 2% of all ischemic strokes but for approximately 20% of strokes in patients younger than 45 years old. Most dissections of extracranial arteries involve some trauma stretch, mechanical stress, or connective tissue abnormalities. In the absence of these disorders, determining the etiology of recurrent extracranial dissections is quite challenging because the underlying nature of these cases is poorly understood. We report the case of a 44-year-old female with recurrent dissections of the vertebral and carotid arteries associated with a heterozygous mutation p.Pro2122Leu in the NOTCH 1 gene. Her mother with a thoracic aortic aneurysm was also positive for this variant.

## Introduction

Dissections of extracranial arteries are estimated to account for only 2% of all ischemic strokes but for approximately 20% of strokes in patients younger than 45 years old (1). Most dissections of extracranial arteries involve some trauma stretch, mechanical stress, or connective tissue abnormalities (1–3). In the absence of these disorders, determining the etiology of recurrent extracranial dissections is quite challenging because the underlying nature of these cases is poorly understood. We report the case of a 44-year-old female with recurrent dissections of the vertebral and carotid arteries associated with a heterozygous mutation p.Pro2122Leu in the NOTCH 1 gene. Her mother with a thoracic aortic aneurysm was also positive for this variant.

## Clinical Case

**Figure 1:**
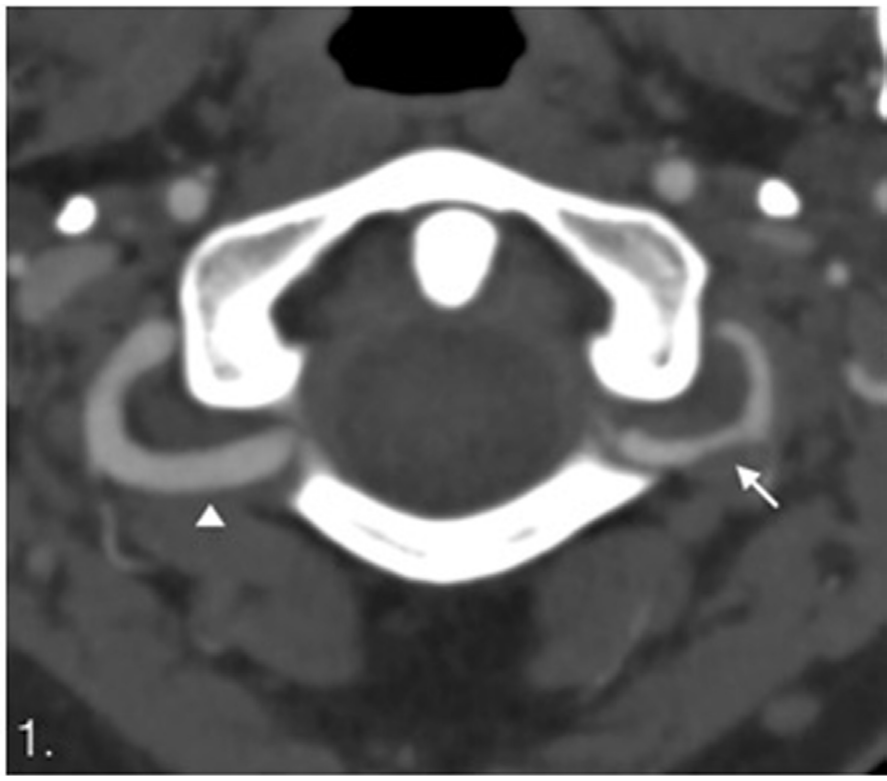
Computed tomography angiography. Dissection of the left vertebral artery with irregular lumen and stenosis (arrow) compare with normal right vertebral artery (arrowhead).

**Figure 2:**
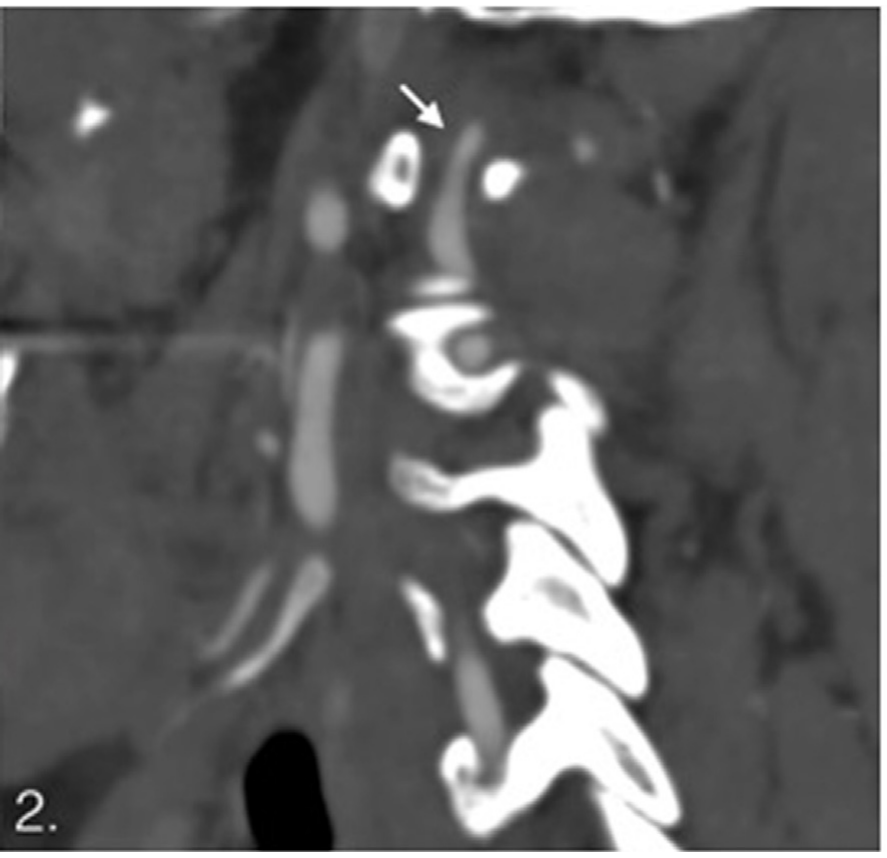
Sagittal computed tomography angiography showing stenosis on V3.

The patient is a 44-year-old female, with a medical history of migraines, mild arterial hypertension, and a left vertebral artery dissection in 2014, without brain infarction (Figures 1 and 2). She was using losartan and aspirin daily. The patient suffers from recurrent headaches related to the first 3 days of her menstruation cycle. Her 75-year-old mother was diagnosed with a thoracic aortic aneurysm 10 years previously.

**Figure 3:**
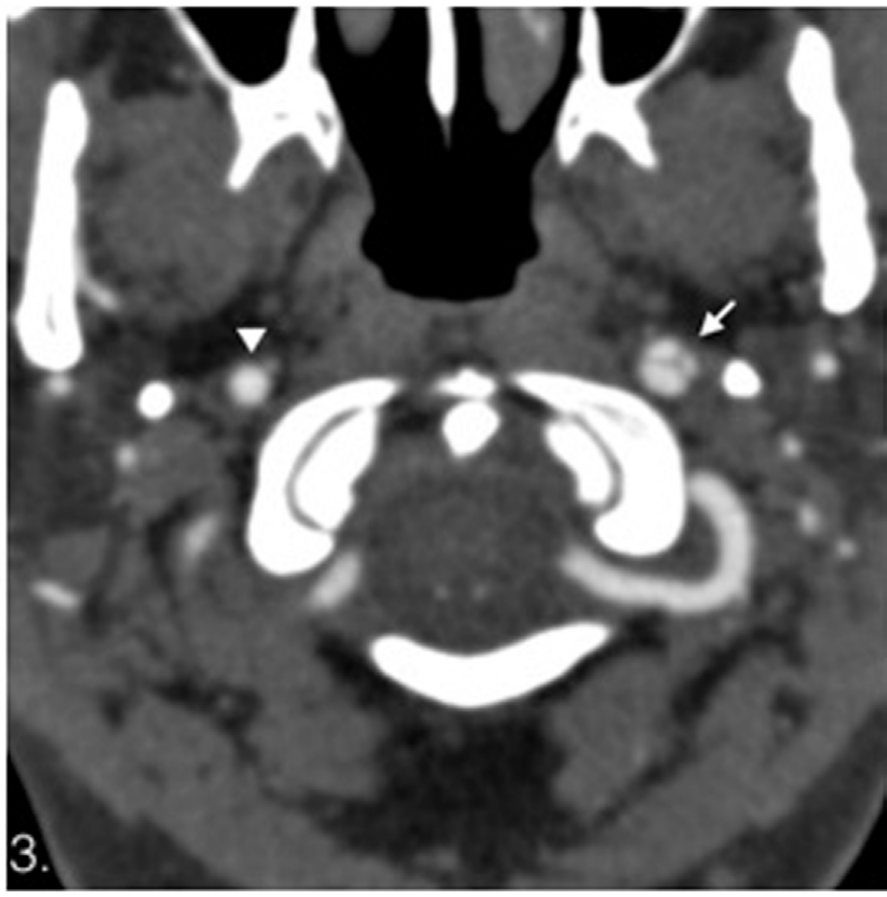
Computed tomography angiography. Dissection of the left internal carotid artery with flaps (white arrow) compare with normal right internal carotid artery (arrowhead). No abnormalities on the left vertebral artery are seen on this study.

**Figure 4:**
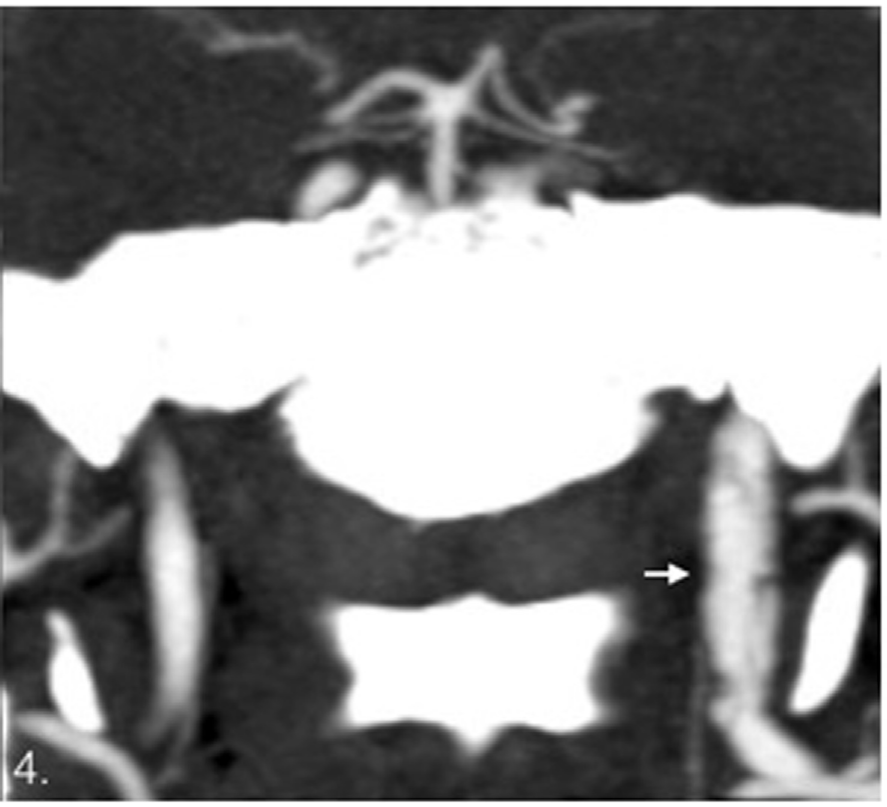
Coronal images. Dissection of the left internal carotid artery with multiple flaps showed as lineal hypodensities in the lumen.

In September 2016, she presented with oppressive headache with left predominance, photophobia and nausea, lasting for 6 days. The intensity of the pain was 8 out of 10 on a visual analog scale (VAS). She also complained of cervical discomfort. A computed tomography angiography (CTA) showed left internal carotid artery dissection extended between the upper cervical and supraclinoid segments (Figures 3 and 4). No ischemic brain damage was observed. Therefore, she was admitted to the emergency department, and aspirin orally was administered in doses of 250 mg at bed time. There were no focal neurological signs on physical examination. The patient had good spatial–temporal orientation and was afebrile.

We attributed the headache to both the left internal carotid artery dissection and the migraine. There was an insufficient response to a combination of ketoprofen and metamizole i.v. Thus, intravenous betamethasone was added to the treatment regimen to provide a rescue medication for the refractory pain. However, the patient showed only a partial response after a 3-day course. She was then prescribed oral topiramate 50 mg two times a day as preventive therapy and naproxen sodium 550 mg as acute treatment option.

One week later, a new CTA was performed and showed no changes compared to the previous imaging.

Apart from hypertension and migraine, we did not identify other putative risk factors for dissections of extracranial arteries such as recent respiratory tract infection and hyperhomocysteinemia. There was no history of major or minor cervical trauma, chiropractic maneuver, or hyperextension injury. Infectious-inflammatory pathology of the paranasal sinuses was excluded with a computed tomography. The patient did not have signs of either hypermobility or dermic lesions. A dermatologist ruled out connective tissue disorders. Renal fibromuscular dysplasia was ruled out using non-invasive imaging duplex ultrasound. Transthoracic echocardiography revealed no abnormalities such as congenital heart defects and left ventricular outflow tract malformations. A computed tomographic scanning and aortography ruled out aortic aneurysm.

To determine the possible nature of this recurrent dissection, an aortopathy comprehensive panel was ordered with sequence analysis and exonic deletion/duplication testing of 25 genes (ACTA2, CBS, COL3A1, COL5A1, COL5A2, EFEMP2, FBN1, FBN2, FLNA; MAT2A, MED12, MYH12, MYH11, MYLK, NOTCH1, PLOD1, PRKG1, SKI, SLCA10, SMAD3, SMAD4, SMAD6, TGFB2, TGFB3, TGBR1, TGFBR2). This test is a comprehensive analysis of genes associated with inherited aortopathy and connective tissue disorders. We ordered it because of the antecedent of maternal thoracic aortic aneurysm (INVITAE, San Francisco, CA, USA; www.invitae.com/en/physician/tests/02301/#test-order).

A heterozygous variant was identified in the NOTCH 1 gene (exon 34, c.6365C>T). This sequence replaces proline with leucine at codon 2122 of the NOTCH 1 protein (p.Pro2122Leu). Her mother was also tested, and she was positive for this variant in heterozygous state.

The patient was discharged without focal neurologic deficit with a National Institutes of Health Stroke Scale (NIHSS) of 0 (0–42). After 6 months, she was on aspirin 100 mg and topiramate 100 mg per day, remained with NIHSS 0 and was able to return to normal activities. There was a reduction in the headache days to about 1 day/month and in the headache severity (VAS = 2).

The study was conducted according to International Standards of Good Clinical Practice (ICH guidelines and the Declaration of Helsinki). The project was approved by the local Research Ethics Committees of Universidad de Chile Hospital, Santiago, Chile. The subject gave written informed consent for the publication of this case report in accordance with the Declaration of Helsinki.

For the genetic definitions, see Table 1.

**Table 1:** Genetic nomenclature.

The Mendelian Inheritance in Man (MIM) number is a numerical assignment for inherited diseases, genes, and functional segments of DNA listed in the comprehensive catalog MIM. More information can be obtained at www.omim.org/ (4)
The Sorting Intolerant From Tolerant Score (SIFT score) is a normalized probability of observing a new amino acid at that position and ranges from 0 to 1. A value of between 0 and 0.05 is predicted to affect protein function. The SIFT platform is available at http://sift.jcvi.org/ and sift.bii.a-star.edu.sg/ (5)
The Polyphen-2 score also ranges from 0 to 1, but in the opposite manner as the SIFT score. A score greater than 0.85–1.0 more confidently predicts protein damage. The Polyphen-2 platform is available at http://genetics.bwh.harvard.edu/pph2/ (6)
Mutation Taster produces outputs such as “disease causing” (probably deleterious), “disease causing automatic” (known to be deleterious), “polymorphism” (probably harmless), and “polymorphism automatic” (known to be harmless). It is available at http://www.mutationtaster.org/ (7)
Exome Aggregation Consortium is a coalition of investigators seeking to aggregate and harmonize exome sequencing data from a wide variety of large-scale sequencing projects and to make summary data available for the wider scientific community. The data set provided on this website spans 60,706 unrelated individuals sequenced as part of various disease-specific and population genetic studies. The information is available at http://exac.broadinstitute.org/about (8)

## Discussion

The patient and her mother with a thoracic aortic aneurysm were positive for the variant p.Pro2122Leu in the NOTCH 1 gene. This finding may help interpret the role of this variant in the pathology of the aorta and extracranial arteries.

The NOTCH 1 gene is located in the locus 9q34.3 and encodes the protein NOTCH 1, which is a member of the NOTCH family of receptors. NOTCH 1 is one of the key genes in early development that regulates many processes such as cell lineage specification, intracellular interactions, regeneration, and apoptosis. This transmembrane receptor protein initiates a signaling pathway that plays an active role in cardiac embryogenesis. This is supported by the fact that mutations in other components of the NOTCH 1 pathway are associated with congenital heart defects and left ventricular outflow tract malformations, including aortic coarctation (9).

The variant p.Pro2122Leu is a rare missense change present in population databases [Exome Aggregation Consortium 0.01% (8)]. Algorithms that were developed to predict the effect of missense changes on protein structure and function all suggest that this variant is likely to be disruptive: Sorting Intolerant From Tolerant Score: 0, damaging; Polyphen-2 score: 0.975, probably damaging; and Mutation Taster: disease causing. These predictions have not been confirmed by published biofunctional studies; therefore, this variant-induced change is of uncertain significance.

Our *in silico* pathogenic prediction for this NOTCH 1 variant is supported by prediction algorithms and the low prevalence of the variant in population databases. Other authors have found some variant of uncertain significance in NOTCH 1 gene, and they suggest that concurrent mutations may be required for phenotype development (10). As a transmembrane receptor, NOTCH 1 protein is more likely to feature phenotypes with heterozygous variants (in just one allele) in comparison with other molecules as the enzymes that need homozygous variants or compound heterozygous variants (a different variant in each allele) to perform pathogenic phenotypes. The variant p.Pro2122Leu changes the last amino acid of the sixth ankyrin repeat motif (ANK) of ankyrin repeat-containing domain located in the intracellular fragment of NOTCH1 (NIC). Each ANK consists of pairs of antiparallel alpha-helices stacked side by side and connected by a series of intervening beta-hairpin (11). Ankyrin repeat-containing domain is conformed by a tandem of ANK, and its function is related to protein–protein interactions. An activation of NOTCH 1 receptor does a cleavage of NIC; then, this structure is translocated into the nucleus binding to CSL, a transcription factor, that turns function from transcriptional repressor to activator when interacts with NIC (12, 13). This different proteic structure in ANK could cause some effect in protein–protein interactions resulting in an impaired function of NIC.

Sequence variants in the NOTCH 1 gene are associated with aortic valve disease-1 [Mendelian Inheritance in Man (MIM): 109730] (14) and Adams–Oliver syndrome-5 (MIM: 616028), a rare developmental disorder defined by aplasia cutis congenita and terminal transverse limb defects that usually features some vascular anomalies and congenital heart defects (15).

A group of spontaneous dissections are related to ultrastructural connective tissue abnormalities, and some disorders are found at a higher rate than expected in patients with arterial dissections [Marfan syndrome (MFS) (MIM: 154700), Ehlers–Danlos syndrome (EDS) classic type (MIM: 13000) and vascular type (MIM: 130050), Loeys–Dietz syndrome (LDS) (MIM: 609192, 610168, 613795, 614816, 615582), and fibromuscular dysplasia of arteries (FMD) (MIM: 135580)].

The transforming growth factor-beta (TGFβ) pathway is involved in MFS, EDS and LDS syndromes (16, 17). There is evidence of an interaction between the NOTCH pathway and TGFβ-2 (18, 19). TGFβ-2 receptor mutations may be involved in thoracic aortic aneurysms in MFS. This is one of the hereditable disorders of the connective tissue associated with aortic root enlargement, mitral valve prolapse and aortic dissections. EDS is also related to mitral valve prolapse, tricuspid valve prolapse, and dilation of the aortic root and pulmonary arteries. LDS has an overlapping featuring with MFS, but LDS presents vascular malformations such as generalized arterial tortuosity. A subgroup of LDS has been explained by variants at TGFβ-3 gene (20).

In stroke, some studies have found associations between an upregulation of the NOTCH1 pathway and brain arteriovenous malformations. The increased expression of NOTCH 1 has been related to hemorrhage of brain arteriovenous malformations (21).

## Concluding Remarks

In summary, the p.Pro2122Leu variant has not previously been reported in the literature in individuals with a NOTCH1-related disease; thus, it has been classified as a variant of uncertain significance. However, its link with developmental pathways of the human cardiovascular system suggests that this variant is likely disruptive in the extracellular matrix of extracranial arteries in non-syndromal extracranial artery dissections.

## Ethics Statement

The study was conducted according to International Standards of Good Clinical Practice (ICH guidelines and the Declaration of Helsinki). The project was approved by the local Research Ethics Committees of Universidad de Chile Hospital, Santiago, Chile.

## Author Contributions

CG—research project: conception, organization, and execution; statistical analysis: design, execution, and review and critique; and manuscript: writing of the first draft and review and critique. GF—review and critique. KB—research project: organization and execution. WS—research project: organization. PA—research project: organization and execution; manuscript: review and critique. MM—manuscript: review and critique. CR—manuscript: writing.

## Conflict of Interest Statement

The authors declare that the research was conducted in the absence of any commercial or financial relationships that could be construed as a potential conflict of interest.
